# Crizotinib-Induced Fulminant Hepatic Failure: A Rare Adverse Event

**DOI:** 10.1200/JGO.2016.007765

**Published:** 2016-12-14

**Authors:** Narayan Adhikari, Pavnesh Kumar, Bhanu P. Venkatesulu, Rambha Pandey, Kunhiparambath P. Haresh, Subhash Gupta, Daya N. Sharma, Goura K. Rath

**Affiliations:** All authors: All India Institute of Medical Sciences, New Delhi, Delhi, India.

## INTRODUCTION

Lung cancer is a leading cause of death with 1.8 million new cases and 1.6 million deaths worldwide in 2012.^1^ About 85% are non–small-cell lung cancer (NSCLC) of which 4% to 7% are positive for anaplastic lymphoma kinase (*ALK*) gene rearrangement.^2-5^ Patients with *ALK* gene mutation define a distinct subgroup who are younger, who are light smokers or nonsmokers, and who have adenocarcinoma histology.^6^ The tyrosine kinase inhibitor crizotinib significantly improved clinical outcome in patients with *ALK*-positive advanced NSCLC when compared with conventional chemotherapy and is currently the first line of treatment for these patients.^7,8^ Crizotinib treatment is commonly associated with an increase in liver aminotransferases, which is reversible on dose reduction or interruption.^9^ We report a case of crizotinib-induced fulminant hepatic failure with hepatic encephalopathy.

## CASE REPORT

A 56-year-old man presented with complaints of left-side chest pain for 2 months and hemoptysis for 1 month. There were no medical comorbidities or familial history of malignancies. The patient was a nonsmoker and occasional drinker. Baseline positron emission tomography and computed tomography (PET/CT) revealed two metabolically active soft tissue masses (one was 2.7 × 2.4 cm in the left suprahilar region and the other was 2.4 × 1.6 cm in the left lower lobe), enlarged prevascular and left hilar lymph nodes, a metastatic lesion in the left fourth rib, and moderate left pleural effusion. Biopsy from the lung mass revealed adenocarcinoma positive for *ALK* gene rearrangement and negative for epidermal growth factor receptor gene mutation by fluorescent in situ hybridization analysis. Pleural fluid cytology was positive for metastatic adenocarcinoma. The diagnosis was advanced NSCLC (T4N2M1a, stage IV, according to the American Joint Committee on Cancer Staging Manual, 7th edition). The baseline hemogram, liver function tests, and kidney function tests were within normal limits.

The patient received palliative radiotherapy with 20 Gy in five fractions over 5 days to the lung mass for controlling hemoptysis. The patient was started on tablet crizotinib 250 mg twice per day; a liver function test (LFT) was recommended once per week for monitoring liver toxicity. After 1 month, PET/CT imaging showed a partial response to therapy with a reduction of more than 30% in the size of primary tumor and a decrease in pleural effusion along with a reduction in uptake of fluorodeoxyglucose. The patient tolerated the treatment well without any significant adverse effects during the first month. Then, after 39 days of crizotinib administration, the patient presented to the emergency department with complaints of generalized weakness, vomiting, poor oral intake, sleep disturbances, and constipation for 2 days. The patient stopped taking crizotinib after the onset of symptoms 2 days before he was hospitalized. A complete blood count and liver and kidney function tests were performed that revealed deranged liver function. Serum bilirubin had increased to 5.2 mg/dL, AST was 96 IU/L, ALT was 64 IU/L, and serum alkaline phosphatase was normal at 238 IU/L. Prothrombin time (PT) was increased to 28.3 seconds, and international normalized ratio (INR) was 2.6. No abnormalities were revealed after a CT scan of the brain was performed. An ultrasonogram of the abdomen did not reveal any focal lesions in the liver or any features of biliary obstruction. Tests were performed for viral markers, including hepatitis B surface antigen, anti-hepatitis C–, anti-hepatitis A–, and anti-hepatitis E–virus antibodies; all were negative thus ruling out viral hepatitis. Serum copper and ceruloplasmin levels were within normal limits. Serum antinuclear antibody and anti–smooth muscle antibody were negative. Tests for cytomegalovirus, Epstein-Barr virus, herpes simplex virus, and HIV were negative. Serum ammonia had increased to 271 μ/dL. Thus, a diagnosis of drug-induced acute liver failure was made according to Hy’s law of drug-induced liver injury.

**Fig 1:**
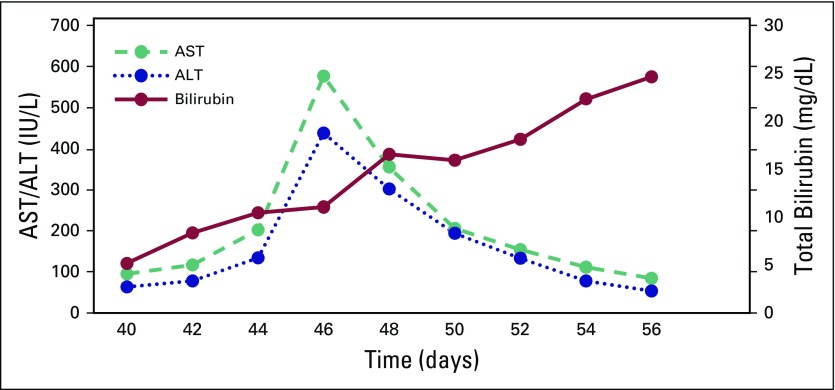
Serial liver enzymes and bilirubin levels with respect to day of crizotinib administration. Day 1 is the first day of crizotinib administration. Crizotinib was stopped on day 39.

**Fig 2:**
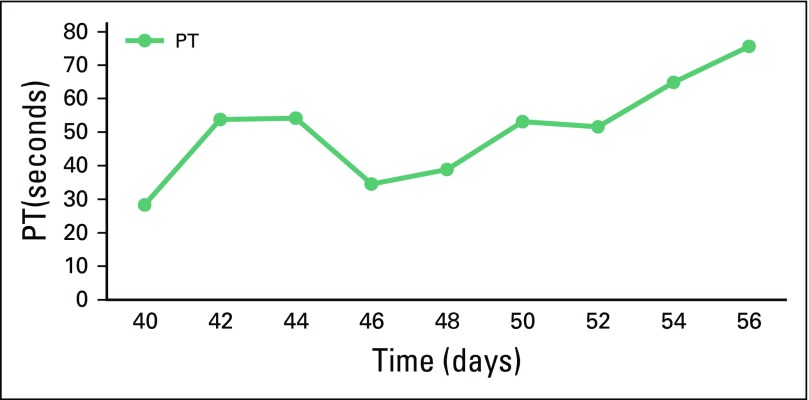
Serial prothrombin time (PT) levels with respect to day of crizotinib administration. Day 1 is the first day of crizotinib administration. Crizotinib was stopped on day 39.

During the course of treatment, the patient developed hepatic encephalopathy, which progressed from grade 2 to grade 4. Fundus examination revealed features suggestive of papilledema. The patient was managed intensively with vitamin K supplementation, fresh frozen plasma transfusion, lactulose enema and laxatives, prophylactic antibiotics, proton pump inhibitors, injectable l-ornithine and l-aspartate, injectable *N*-acetylcysteine, and mannitol, according to guidelines from the American Association for the Study of Liver Diseases. The option of liver transplantation was ruled out in view of his metastatic disease. The patient worsened clinically with progressive deterioration in functional status. Bilirubin, PT /INR, and hepatic aminotransferase enzyme level fluctuations are depicted in Figures 1 and 2. The patient died after 18 days of hospitalization as a result of multiorgan dysfunction.

## DISCUSSION

Crizotinib is a tyrosine kinase inhibitor of ALK, MET, and ROS1 kinases.^10^ Commonly reported adverse effects include nausea, vomiting, diarrhea, constipation, fatigue, transient visual disorders, peripheral edema, and neutropenia. Potentially serious adverse effects include interstitial lung disease and QT prolongation. Grade 3 to 4 increases in aminotransferases were observed in few patients.^11^ Crizotinib was reported to cause a grade 3 increase of liver enzymes in 14% to 16% of patients in two phase III trials; a majority of these symptoms were reversible on dose interruption.^7,8^ One patient had fatal acute liver failure after safety data closure.^7^ Two cases of fatal hepatic failure and one case of reversible hepatitis have been previously reported in the literature.^11-13^ Therefore, LFT monitoring once per week is recommended during the first 2 months after starting crizotinib and monthly thereafter, according to prescribing information.^14^ This is the third case of crizotinib-induced fulminant hepatic failure with hepatic encephalopathy reported in the literature. We have ruled out other causes of liver failure such as viral, biliary obstruction, alcoholic, autoimmune, and metabolic causes.

Ripault et al^13^ reported on the first patient with crizotinib-induced acute hepatitis, who relapsed after reintroduction of the drug. But LFTs returned to normal after the drug was discontinued, even in the recurrent setting. Sato et al^12^ reported the first case of a fatal outcome. A 54-year-old woman with a history of hepatitis C infection who had started crizotinib was hospitalized on day 29 of crizotinib administration with increased liver enzymes. She was managed intensively with plasma exchange, continuous hemodiafiltration, and high-dose steroid therapy. But she did not respond to therapy and died on day 36. Van Geel et al^11^ reported on the second patient who presented with liver failure on day 24 of crizotinib therapy. She was treated intensively by following Dutch guidelines for liver failure and encephalopathy, but she ultimately died on day 40.

Our patient received crizotinib for a total of 39 days before the appearance of symptoms and progression to liver failure. Despite the discontinuation of the drug and intensive management, his condition worsened. He died on day 57, 18 days after crizotinib was discontinued. The exact mechanism of crizotinib-induced hepatotoxicity is unknown. But sporadic, dose-independent fulminant liver failure, as our patient experienced, suggests the possibility of immune mechanisms. Our case highlights the possibility of acute liver injury in patients treated with crizotinib, even with strict monitoring of liver functions. The weekly monitoring of liver enzymes may not be adequate to prevent these sporadic cases with fatal outcome. Future research should be directed toward establishing the mechanism of crizotinib-induced liver injury and identifying the potential risk factors for fulminant liver failure triggered by crizotinib. In the literature, various factors that may predispose to more severe hepatotoxicity by crizotinib have been proposed, including CYP3A inducers or inhibitors, a previous history of hepatitis C virus infection, antidiabetic drugs, or collagen disorders.^12^ But no direct cause-effect relationship has been established. Our patient did not have any of these associated factors.

Although crizotinib is usually well tolerated, except for some mild and reversible adverse effects, physicians should acknowledge the possibility of irreversible fulminant hepatotoxicity. Identification and surveillance for the potential risk factors should be endorsed in future research. In routine practice, LFTs should be performed once per week for the first 2 months of treatment and more frequently if subtle derangement of liver enzymes or associated comorbidities are observed.
